# Pediatric endoscopic mucosal resection: A 10‐year single‐center experience

**DOI:** 10.1002/jpn3.70194

**Published:** 2025-08-12

**Authors:** Brett J. Hoskins, Jared M. Grabau, Douglas K. Rex

**Affiliations:** ^1^ Department of Pediatrics, Division of Pediatric Gastroenterology, Hepatology, and Nutrition Indiana University School of Medicine, Riley Hospital for Children at IU Health Indianapolis Indiana USA; ^2^ Department of Medicine Marian University Tom and Julie Wood College of Osteopathic Medicine Indianapolis Indiana USA; ^3^ Department of Medicine, Division of Gastroenterology and Hepatology Indiana University School of Medicine Indianapolis Indiana USA

**Keywords:** conventional EMR, pediatric endoscopy, polypectomy, submucosal injection

## Abstract

**Objectives:**

Endoscopic mucosal resection (EMR) is well established in adult gastroenterology but remains underutilized in pediatrics due to limited data, training opportunities, and equipment. This study presents a 10‐year, single‐center experience with conventional hot and cold snare EMR, band‐assisted (B‐EMR), and underwater EMR (U‐EMR) techniques in pediatric patients.

**Methods:**

A retrospective review was conducted of all EMR procedures performed in patients under 21 years of age between 2015 and 2025 at a tertiary care children's hospital. Data on patient demographics, lesion characteristics, procedural details, pathology, and outcomes were collected and analyzed descriptively.

**Results:**

Twenty EMRs were performed in 18 patients (mean age 17.1 years, range 3–20). The most common underlying diagnoses included familial adenomatous polyposis (*n* = 7), sporadic mucosal polyps (*n* = 4), subepithelial lesions (*n* = 4), juvenile polyposis syndrome (*n* = 2), Peutz‐Jeghers syndrome (*n* = 1), and Lynch syndrome (*n* = 1). Lesions ranged from 6 to 80 mm and were located throughout the gastrointestinal tract, most commonly in the colon (*n* = 9), duodenum (*n* = 5), and esophagus (*n* = 3). Techniques included hot snare EMR (*n* = 9), cold snare EMR (*n* = 6), B‐EMR (*n* = 4), and U‐EMR (*n* = 1). Complete resection was achieved in 95% of cases, with one incomplete resection requiring surgical management for adenocarcinoma. B‐EMR was safely applied to subepithelial lesions. No delayed complications occurred.

**Conclusions:**

EMR is feasible, safe, and effective in pediatric patients for both mucosal and subepithelial lesions. Broader adoption in pediatric practice will require expanded training, multidisciplinary collaboration, and development of pediatric‐specific guidelines. These findings support EMR as a valuable therapeutic option in complex pediatric gastrointestinal disease.

## Graphical abstract

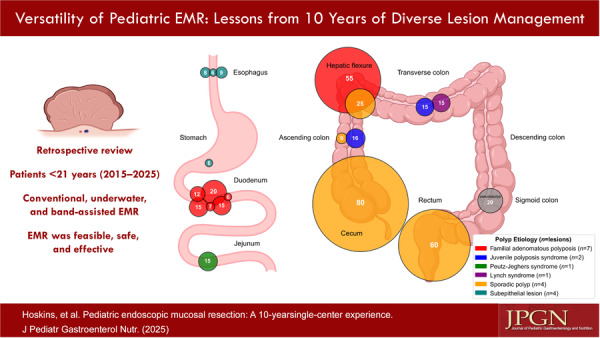

## 1 INTRODUCTION

Endoscopic mucosal resection (EMR) is a minimally invasive technique used to remove superficial gastrointestinal lesions. Conventional EMR involves submucosal injection followed by snare resection and is primarily utilized for the treatment of flat or large sessile polyps, dysplastic lesions, and intramucosal carcinoma. In adult gastroenterology, EMR has become a cornerstone of therapeutic endoscopy, offering curative potential while avoiding the higher morbidity, mortality, and cost associated with surgical resection.^1^ According to the US Multi‐Society Task Force on Colorectal Cancer, EMR is the preferred treatment for large (≥ 20 mm) nonpedunculated colorectal lesions and may be performed en bloc or in a piecemeal fashion depending on lesion size and location.^2^

In pediatrics, EMR remains underutilized despite growing interest in its application.^3^, ^4^ This is particularly relevant in hereditary polyposis syndromes such as familial adenomatous polyposis (FAP), juvenile polyposis syndrome (JPS), and Peutz‐Jeghers syndrome (PJS), which often present during childhood or adolescence.^5^ Unlike adult populations—where EMR is frequently needed for large sessile lesions and early mucosal neoplasia—such indications are less commonly encountered in children, contributing to slower adoption of the technique in pediatric practice. Limited pediatric‐specific data, reduced procedural volume, equipment constraints, and lack of therapeutic endoscopy training opportunities have further hindered its widespread use.^3^ Nonetheless, emerging reports—including two retrospective reviews and three case reports—suggest that EMR can be performed safely and effectively in children and adolescents.^3^, ^4^

Beyond mucosal lesions, pediatric endoscopists may also encounter subepithelial gastrointestinal lesions, historically managed with surgery or conservative surveillance. In adult practice, alternative EMR techniques such as band‐assisted EMR (B‐EMR) and underwater EMR (U‐EMR) have been increasingly been used for both mucosal and subepithelial lesions, including when conventional submucosal lifting is limited by lesion morphology or fibrosis.^6^, ^7^ These techniques also offer practical solutions for challenging anatomic locations such as the duodenum, esophagus, and ampullary region. Pediatric data on these techniques remain virtually absent.

This 10‐year retrospective review presents a comprehensive pediatric EMR experience at a single tertiary center, encompassing conventional hot and cold snare EMR, B‐EMR, and U‐EMR. Our findings expand the current pediatric EMR literature by reporting outcomes for both mucosal and subepithelial lesions across a wide range of gastrointestinal locations. We also address procedural considerations, identify knowledge gaps, and propose future directions to better define EMR's evolving role in the management of gastrointestinal lesions in children and adolescents.

## 2 METHODS

### 2.1 Ethics statement

This study was approved by the Indiana University School of Medicine Institutional Review Board (#26141) and conducted in accordance with institutional policies. Informed consent was waived due to the retrospective design.

### 2.2 Study design, setting, and population

We conducted a retrospective review of all EMR procedures performed in patients under 21 years of age across the Indiana University Health system between March 2015 and March 2025. All patients under 21 who underwent EMR during the study period were included. No cases were excluded based on indication or underlying diagnosis.

### 2.3 Data collection

Cases were identified using CPT codes 43254 (esophagogastroduodenoscopy with EMR) and 45390 (flexible colonoscopy with EMR). Abstracted data included demographics, indication for EMR, lesion location, size, morphology, resection technique (hot snare EMR [HS‐EMR], cold snare [CS‐EMR], B‐EMR, or U‐EMR), method of removal (en bloc or piecemeal), injection solution, completeness of resection, pathology, and complications.

### 2.4 Statistical analysis

Descriptive statistics were used. Continuous variables were summarized as means or ranges; categorical variables were reported as counts and percentages. Due to the small sample size and descriptive scope, no inferential statistical analyses were performed.

### 2.5 EMR procedure

All EMRs were performed in hospital‐affiliated endoscopy units across both inpatient and outpatient settings. Following lesion identification, submucosal injections were used to achieve adequate mucosal lift for HS‐EMR or CS‐EMR techniques. Injection agents included normal saline with dye or commercial lifting agents. B‐EMR and U‐EMR were performed without injection. B‐EMR was primarily used for small subepithelial lesions in the esophagus and gastric antrum with a band ligation device. U‐EMR was used in the colon to facilitate resection via water immersion. Resection was performed with snares, with or without electrocautery, and specimens were retrieved for histology. Lesions were removed en bloc when feasible; otherwise, piecemeal resection was performed. Hemostasis was achieved with clips, cautery, or hemostatic gel as needed. All patients underwent post‐procedural monitoring to assess for immediate complications.

### 2.6 Video demonstrations

Supplemental videos illustrate representative EMR techniques outside this cohort. HS‐EMR is demonstrated with piecemeal resection of a granular laterally spreading tumor (LST‐G) in the cecum (Video S1A), en bloc resection of a large flat cecal polyp with final defect appearance following clip closure (Video S1B), and clip closure of a defect using the zipper technique (Video S1C). U‐EMR is shown for en bloc resection of a large sessile rectal polyp (Video S2A) and for a large flat, nongranular transverse colon lesion in which a submucosal tattoo prompted use of U‐EMR due to concern for fibrosis (Video S2B).

## 3 RESULTS

### 3.1 Patient and lesion overview

Eighteen pediatric patients (mean age 17.1 years, range 3–20; 7 females and 11 males) underwent 20 EMR procedures for mucosal or subepithelial gastrointestinal lesions. The most common underlying condition was FAP (*n* = 7), followed by sporadic polyps (*n* = 4), subepithelial lesions (*n* = 4), JPS (*n* = 2), PJS (*n* = 1), and Lynch syndrome (*n* = 1). One case involved EMR for a large pseudopolyp in the setting of Crohn's disease (*n* = 1).

Lesions were distributed throughout the gastrointestinal tract. The most common sites were the colon (*n* = 9; hepatic flexure, ascending, transverse, sigmoid, and rectum), duodenum (*n* = 6), and esophagus (*n* = 3). Other sites included the jejunum (*n* = 1) and gastric antrum (*n* = 1). Lesion sizes ranged from 6 to 80 mm. Morphologies included sessile (*n* = 8), flat (*n* = 7), semi‐pedunculated (*n* = 4), and oval (*n* = 2), with some showing multi‐lobulation or fibrosis.

**Figure 1:**
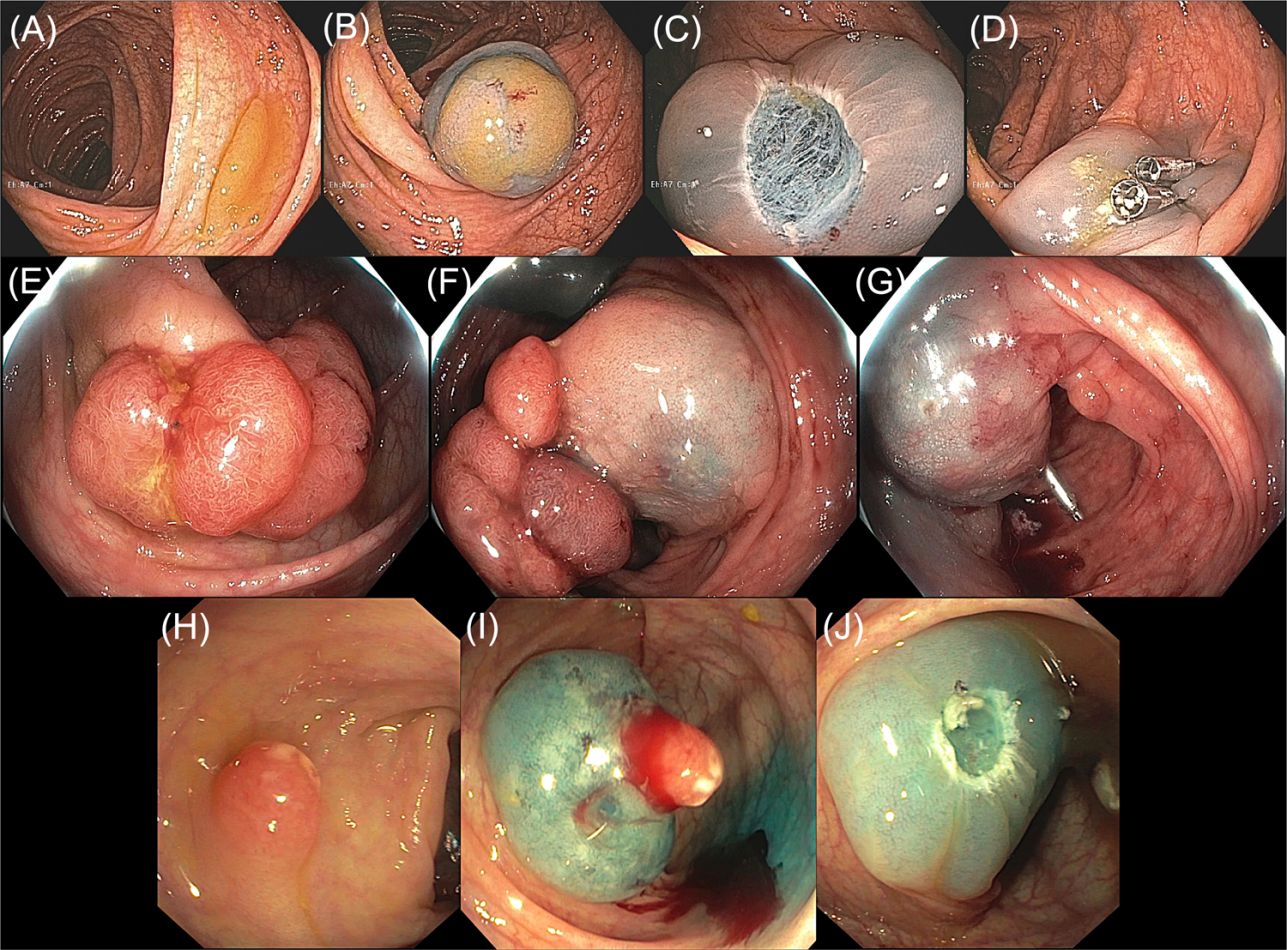
Representative cases of HS‐EMR used for polyp removal in pediatric patients. (A–D) Flat polyp in the ascending colon of a 20‐year‐old patient, shown presubmucosal injection (A), postlifting (B), following en bloc resection (C), and after clip closure of the mucosal defect (D). (E–G) Semi‐pedunculated polyp in the hepatic flexure of a 17‐year‐old patient, shown presubmucosal lifting (E), postlifting (F), and following piecemeal resection with clip closure (G). (H–J) Semipedunculated polyp in the transverse colon of a 5‐year‐old patient, shown presubmucosal lifting (H), postlifting (I), and following en bloc resection (J). HS‐EMR, hot snare endoscopic mucosal resection.

**Figure 2:**
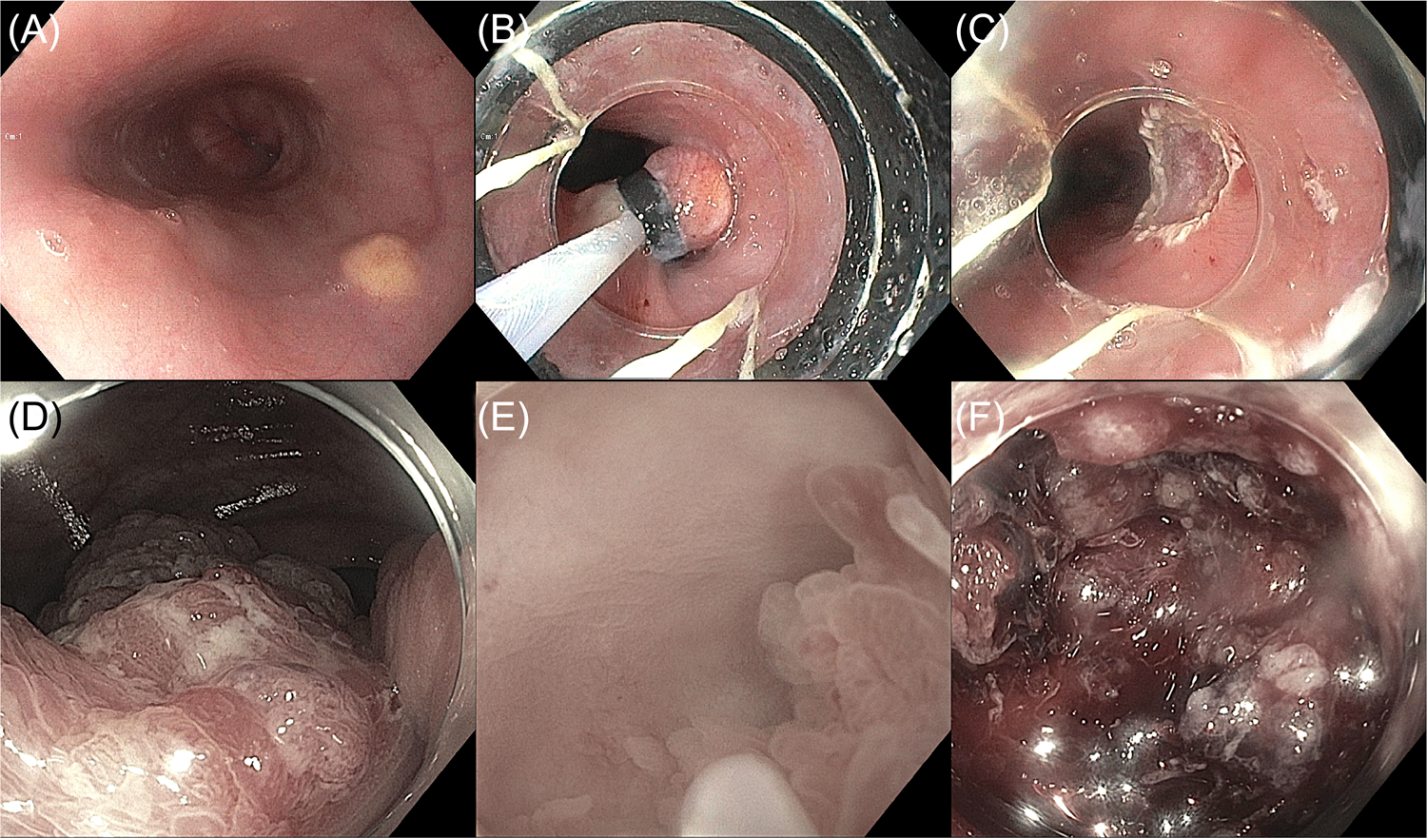
Representative cases of B‐EMR and U‐EMR for polyp removal in pediatric patients. (A–C) Subepithelial lesion in the esophagus of a 20‐year‐old patient, shown before resection (A), after band deployment and snare placement (B), and following en bloc removal (C) using B‐EMR. (D–F) Large sessile polyp in the cecum of a 20‐year‐old patient, shown before resection (D), during underwater EMR (E), and complicated by postresection bleeding (F). Complete endoscopic resection was not feasible due to significant fibrosis. B‐EMR, band‐assisted endoscopic mucosal resection; EMR, endoscopic mucosal resection; U‐EMR, underwater endoscopic mucosal resection.

**Figure 3:**
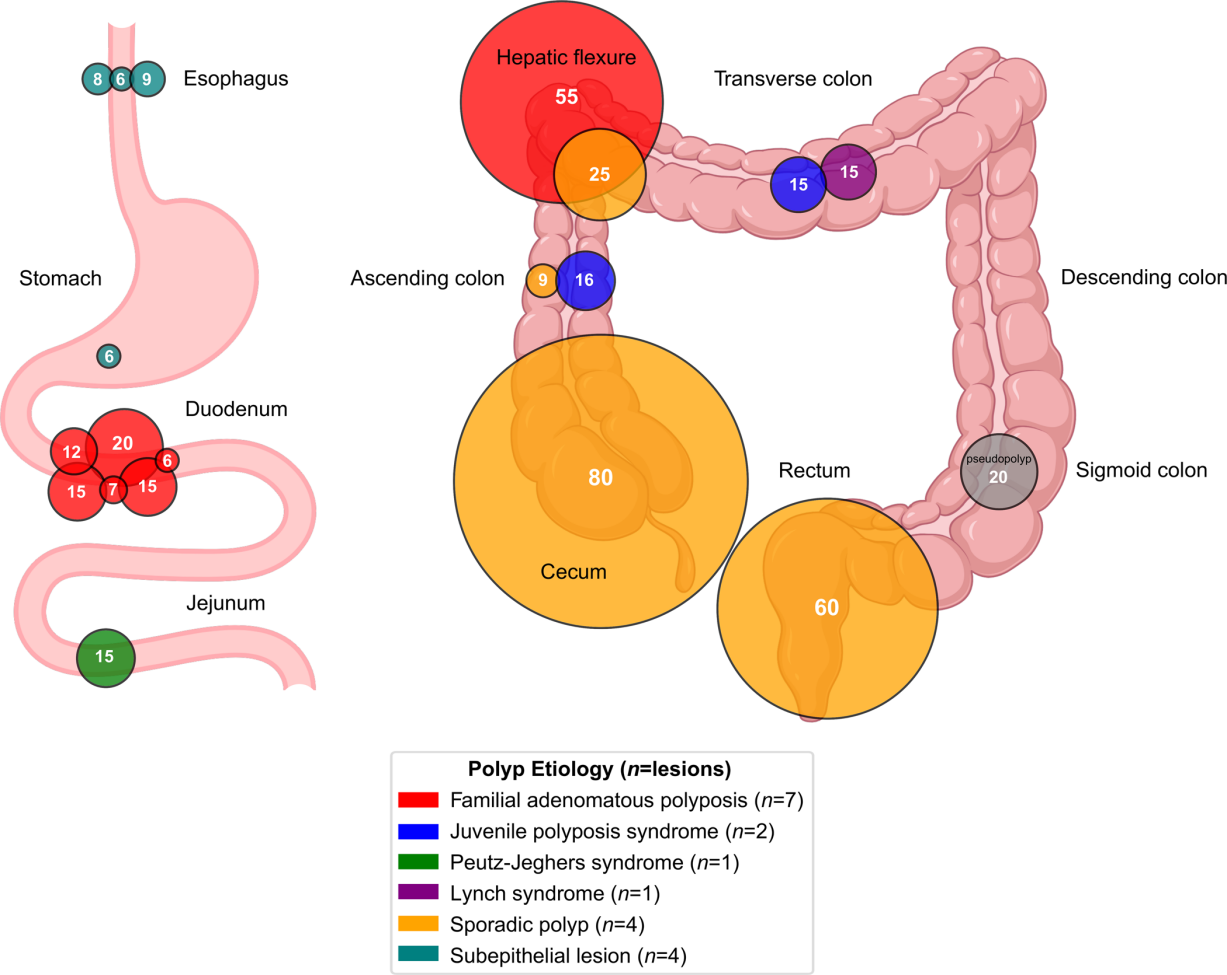
Anatomic distribution and size of resected polyps during pediatric EMR. Each circle corresponds to a polyp showing size comparison, with the number inside the circle indicating lesion size in millimeters (mm). Diagram credit: BioRender. Hoskins, BJ. (2025). https://biorender.com/qayjo0n. EMR, endoscopic mucosal resection.

Table S1 summarizes clinical and procedural details. Figures 1 and 2 showcase EMR cases from the study cohort. Specifically, Figure 1 demonstrates the use of HS‐EMR, while Figure 2 depicts applications of B‐EMR and U‐EMR. Figure 3 illustrates the anatomic distribution of polyps according to the size and etiology.

### 3.2 EMR techniques and injection solutions

Techniques included HS‐EMR (*n* = 9), CS‐EMR (*n* = 6), B‐EMR (*n* = 4), and U‐EMR (*n* = 1). Injection solutions included saline with methylene blue (*n* = 6); EverLift® (*n* = 6; GI Supply, Inc.); HEXTEND® with methylene blue (*n* = 1; Hospira, Inc., a Pfizer company); ORISE® (*n* = 1; Boston Scientific Corporation); and saline with indigo carmine (*n* = 1). Four B‐EMRs for subepithelial lesions and one U‐EMR were performed without the use of submucosal injection.

### 3.3 Resection and outcomes

En bloc resection was achieved in 13 cases (65%) and piecemeal in 7 (35%). Only one case was incompletely resected due to significant fibrosis—a large 80 mm sessile cecal polyp, attempted with U‐EMR (Figure 2D–F). Subsequent surgical hemicolectomy revealed adenocarcinoma arising from a tubulovillous adenoma with high‐grade dysplasia. In that case, bleeding occurred intraprocedurally and was controlled using hemostatic clips and hemostatic gel. No delayed complications occurred in any case.

Histologic findings included adenomas (*n* = 10; tubular [*n* = 4], sessile serrated [*n* = 1], tubulovillous with high‐grade dysplasia and adenocarcinoma [*n* = 1], and not further classified [*n* = 4]), juvenile polyps (*n* = 2), hamartomatous polyps (*n* = 2), hyperplastic polyp (*n* = 1), pseudopolyp (*n* = 1), and subepithelial lesions including granular cell tumors (*n* = 3) and ectopic pancreas (*n* = 1).

## 4 DISCUSSION

This single‐center retrospective study demonstrates the feasibility, safety, and clinical utility of EMR for managing mucosal and subepithelial lesions in children and adolescents. We report outcomes for multiple EMR techniques across diverse lesion types and locations, expanding the limited pediatric EMR literature.

Several cases illustrate the critical therapeutic role of EMR in pediatrics. Multiple duodenal adenomas were resected in a teenager with FAP using CS‐EMR without complication. A 3‐year‐old with JPS underwent successful HS‐EMR for a semi‐pedunculated colonic polyp. An 11‐year‐old with PJS had a large flat jejunal hamartomatous polyp resected during enteroscopy. In one 20‐year‐old with a large sessile colonic lesion, incomplete resection due to fibrosis revealed adenocarcinoma on surgical pathology (Figure 2D–F). These cases emphasize EMR's versatility across polyp types and locations, and the importance of distinguishing lesions amenable to EMR from those requiring surgery.

In pediatric polyposis syndromes, a variety of polyp morphologies are encountered, including pedunculated, sessile, semipedunculated, and flat lesions.^5^, ^8^ EMR is particularly useful for large sessile, flat, and semipedunculated polyps, enabling complete resection while preserving surrounding mucosa and reducing the need for bowel surgery.^3^, ^4^ In contrast, adult EMR practice often centers on large LSTs, intramucosal carcinoma, or serrated lesions requiring piecemeal or en bloc resection.^2^ These differences highlight the need for pediatric‐specific training that accounts for variation in lesion type, size, anatomy, and technical considerations.

This series also provides insights into resection technique selection. HS‐EMR remains the most commonly used modality, offering clean histologic margins and effective cauterization, but with higher risk of delayed bleeding and thermal injury.^9^ CS‐EMR avoids electrocautery‐related risks and is advantageous in the small bowel, where thermal injury could cause perforation. Its drawback is less distinct histologic margins, possibly increasing the risk of recurrence.^10^ Although immediate bleeding occurs more frequently with CS‐EMR,^10^ it is usually minor and self‐limited. U‐EMR, a newer technique, was infrequently used in this series. A recent trial showed higher en bloc resection rates with U‐EMR compared to conventional EMR, though recurrence rates were similar.^11^ In our study, U‐EMR was attempted for a large, fibrotic cecal lesion but did not achieve complete resection. B‐EMR was safely used for small subepithelial lesions such as granular cell tumors and ectopic pancreas, with complete resection and no adverse events—mirroring outcomes in adult studies.^12^

Several limitations warrant consideration. The small sample size reflects the rarity of complex resectable lesions in children and limited use of EMR in pediatric practice. As a single‐center retrospective study, data are subject to referral and selection bias, limiting generalizability. Surveillance intervals and recurrence data were variable and not systematically assessed. Additionally, the lack of pediatric‐specific tools and formal training opportunities continues to necessitate adaptation of adult techniques and reliance on a small number of experienced proceduralists.

As pediatric gastroenterologists increasingly manage complex mucosal and subepithelial lesions, there is a growing need to expand training in advanced resection techniques. The American Society for Gastrointestinal Endoscopy (ASGE) currently offers intensive hands‐on introductory and advanced EMR courses and its guidelines support adapting endoscopic techniques—including equipment and training—for pediatric use. Formal pediatric EMR training remains limited, prompting many endoscopists to gain experience through apprenticeship‐style learning with adult colleagues. In this series, procedures were performed by both pediatric and adult therapeutic endoscopists. The pediatric endoscopist (B.J.H.) completed ASGE's advanced EMR course and additional apprenticeship‐based training, illustrating one pathway for acquiring proficiency in pediatric EMR. Such collaborative training is essential given the technical nuances, anatomic variability, and risk profiles in children. These factors reflect the complexity of pediatric EMR and the need for continued advancement in training and technique.

## 5 CONCLUSION

This 10‐year experience highlights EMR as a safe, versatile tool for managing complex pediatric polyps. Integration into pediatric practice requires collaboration with adult endoscopists, investment in training, and pediatric specific research. EMR is poised to become a vital part of therapeutic pediatric endoscopy as experience, training, and innovation continue to evolve.

## CONFLICTS OF INTEREST STATEMENT

Brett J. Hoskins: Consultantship—Mirum Pharmaceuticals, Inc. and 3‐D Matrix, Inc. Research Support—Travere Therapeutics, Inc. and Mirum Pharmaceuticals, Inc. Douglas K. Rex: Consultantship—Aries Pharmaceuticals, Inc., Boston Scientific Corporation, Olympus Corporation, Braintree Laboratories, Inc., Sebela Pharmaceuticals, Inc. Research Support—Boston Scientific Corporation, Sebela Pharmaceuticals, Inc., Medtronic plc, Olympus Corporation, Erbe Elektromedizin GmbH. Ownership Interest—Satisfai Health Inc. The remaining author declares no conflict of interest.

## Supporting information

Supporting information.

**Supplemental Video 1A.** Hot snare EMR (HS‐EMR) performed for en bloc resection of a granular laterally spreading tumor (LST‐G) in the cecum. Submucosal injection with lifting agent is followed by snare resection with electrocautery, achieving complete removal.

**Supplemental Video 1B.** Hot snare EMR (HS‐EMR) performed for en bloc resection of a large flat polyp in the cecum. The video demonstrates the resection itself and the final appearance after defect closure. Submucosal injection and the clip deployment process are not shown.

**Supplemental Video 1C.** Clip closure of a mucosal defect following hot snare EMR (HS‐EMR) using the zipper technique. Sequential application of through‐the‐scope clips achieves complete linear closure of the post‐resection defect.

**Supplemental Video 2A.** Underwater EMR (U‐EMR) of a large sessile polyp in the distal rectum. The lesion is removed en bloc without submucosal injection using water immersion to facilitate snare capture and resection.

**Supplemental Video 2B.** Underwater EMR (U‐EMR) of a large flat, non‐granular lesion in the transverse colon. En bloc resection is achieved using water immersion. A submucosal tattoo placed beneath the polyp by the referring physician is visible during resection, which prompted use of U‐EMR in this case due to concern for submucosal fibrosis.
