# Supplementary material for: Pediatric endoscopic mucosal resection: A 10‐year single‐center experience
Source: J Pediatr Gastroenterol Nutr. 2025 Aug 12;81(5):1311–7. doi: 10.1002/jpn3.70194 (PMC12580460; doi:10.1002/jpn3.70194)
Supplement: Supplementary file 1 — Supporting information. [file JPN3-81-1311-s001.docx]

| Patient | Age (years) | Gender | Underlying Condition | Lesion Location | Lesion Size* (mm) | Morphology | EMR Technique | Endoscopist | Removal | Resection Completeness** | Injection Solution*** | Pathology | Complications |
| --- | --- | --- | --- | --- | --- | --- | --- | --- | --- | --- | --- | --- | --- |
| 1 | 17 | Male | FAP | Hepatic flexure | 55 | Semi-pedunculated;  Multi-lobulated | HS-EMR | Pediatric | Piecemeal | Complete | EverLift | Tubular adenoma | None |
| 2 | 18 | Male | FAP | Duodenal ampulla | 15 | Sessile | HS-EMR | Adult | En bloc | Complete | Saline + methylene blue | Adenoma | None |
| 3 | 19 | Male | FAP | Duodenum | 6 | Flat | CS-EMR | Adult | En bloc | Complete | Saline + methylene blue | Adenoma | None |
| 3 | 19 | Male | FAP | Duodenum | 7 | Flat | CS-EMR | Adult | En bloc | Complete | Saline + methylene blue | Adenoma | None |
| 4 | 19 | Male | FAP | Duodenal ampulla | 12 | Sessile | HS-EMR | Adult | En bloc | Complete | Saline + methylene blue | Tubular adenoma;  Low-grade dysplasia | None |
| 5 | 20 | Female | FAP | Duodenum | 20 | Flat | CS-EMR | Adult | En bloc | Complete | EverLift | Adenoma | None |
| 6 | 20 | Female | FAP | Duodenum | 15 | Sessile | CS-EMR | Adult | Piecemeal | Complete | EverLift | Tubular adenoma | None |
| 7 | 3 | Male | JPS | Ascending colon | 16 | Semi-pedunculated | HS-EMR | Pediatric | En bloc | Complete | EverLift | Juvenile polyp | None |
| 8 | 5 | Male | JPS | Transverse colon | 15 | Semi-pedunculated | HS-EMR | Pediatric | En bloc | Complete | EverLift | Juvenile polyp | None |
| 9 | 11 | Male | PJS | Jejunum | 15 | Flat/irregular | CS-EMR | Pediatric | Piecemeal | Complete | EverLift | Hamartomatous polyp | None |
| 10 | 17 | Female | Lynch syndrome | Transverse colon | 15 | Sessile | HS-EMR | Pediatric | Piecemeal | Complete | Saline + indigo carmine | Tubular adenoma | None |
| 11 | 18 | Male | Sporadic polyp | Rectum | 60 | Semi-pedunculated;  Multi-lobulated | HS-EMR | Adult | Piecemeal | Complete | Saline + methylene blue | Hamartomatous polyp | None |
| 12 | 19 | Female | Sporadic polyp | Hepatic flexure | 25 | Sessile | HS-EMR | Adult | En bloc | Complete | HEXTEND + methylene blue | Sessile serrated adenoma | None |
| 13 | 20 | Female | Sporadic polyp | Ascending colon | 9 | Flat | HS-EMR | Adult | En bloc | Complete | Saline + methylene blue | Hyperplastic polyp | None |
| 14 | 20 | Male | Sporadic polyp | Cecum | 80 | Sessile | U-EMR | Adult | Piecemeal | Incomplete due to fibrosis | N/A | Tubulovillous adenoma;  High-grade dysplasia; Adenocarcinoma | Immediate bleeding with successful hemostasis |
| 15 | 19 | Male | Crohn’s disease | Sigmoid colon | 20 | Sessile | CS-EMR | Adult | Piecemeal | Complete | ORISE | Pseudopolyp | None |
| 16 | 16 | Male | Subepithelial lesion | Esophagus | 8 | Oval | B-EMR | Adult | En bloc | Complete | N/A | Granular cell tumor | None |
| 17 | 20 | Female | Subepithelial lesion | Gastric antrum | 6 | Oval | B-EMR | Adult | En bloc | Complete | N/A | Ectopic pancreas | None |
| 18 | 20 | Female | Subepithelial lesion | Esophagus | 6 | Flat | B-EMR | Adult | En bloc | Complete | N/A | Granular cell tumor | None |
| 18 | 20 | Female | Subepithelial lesion | Esophagus | 9 | Flat | B-EMR | Adult | En bloc | Complete | N/A | Granular cell tumor | None |

**Table 1. Summary of Pediatric Endoscopic Mucosal Resection Cases and Outcomes.
***Lesion size reflects maximum dimension.
******Histologic margin assessment is not possible in piecemeal resections; completeness refers to endoscopic impression of full removal.
*******Commercially available injection solutions used included EverLift® (GI Supply), ORISE® (Boston Scientific), and HEXTEND® (Pfizer). **Abbreviations:** B-EMR, band-assisted endoscopic mucosal resection; CS-EMR, cold snare endoscopic mucosal resection; EMR, endoscopic mucosal resection; FAP, familial adenomatous polyposis; HS-EMR, hot snare endoscopic mucosal resection; JPS, juvenile polyposis syndrome; PJS, Peutz-Jeghers syndrome; U-EMR, underwater endoscopic mucosal resection.
